# Comparing School Lunch and Canteen Foods Consumption of Children in Kayseri, Turkey

**DOI:** 10.12669/pjms.303.4651

**Published:** 2014

**Authors:** Dilek Ongan, Neriman Inanc, Betül Cicek

**Affiliations:** 1 Dilek Ongan, Assistant Professor, Department of Nutrition and Dietetics, Izmir Katip Celebi University, Izmir, Turkey.; 2 Neriman Inanc, Professor, Department of Nutrition and Dietetics Nuh Naci Yazgan University, Kayseri, Turkey.; 3 Betül Cicek, Associate Professor, Department of Nutrition and Dietetics; 4 Erciyes University, Kayseri, Turkey.

**Keywords:** School lunch, School aged children, Nutrition

## Abstract

***Objective: ***School Nutrition Programs (SNPs) may have positive effects on children’s food choices through high nutritional quality meals. This cross-sectional & descriptive study was conducted to determine nutritional quality of school lunch and to compare lunch consumption of students who participated in SNP and who did not, at the first governmental school serving school lunch in Kayseri, Turkey.

***Methods: ***One hundred and sixteen students aged 9-14 years were divided into two groups after being matched according to gender, age, grade; 58 participants (school lunch group; SL-G) and 58 nonparticipants (school canteen group; SC-G) were recruited. Energy-nutrient content of 5-day school lunch was determined by recipes. Socio-demographic data and lunch consumption on 5 consecutive weekdays with weighed left overs were obtained. Lunch energy-nutrient intakes and anthropometric measurements were compared.

***Results: ***School lunch was adequate for vitamins (E & C), fibre, iron, inadequate for energy, carbohydrate, folate, calcium. Contribution of fat (36.6±6.8%) and saturated fat (12.2±3.5%) to energy and sodium content was high (1001 mg) in school lunch. SL-G consumed significantly higher protein, vitamin C, thiamine, vitamin B_6_, potassium, magnesium, iron, zinc (p<0.001 for each) than SC-G. Energy (p<0.001), carbohydrate (p<0.001), fat (p<0.05), vitamin E (p<0.001) intakes of SC-G were significantly higher than SL-G. Body weights, height, body mass index of groups were similar.

***Conclusions:*** Foodservice at school should be revised with collaboration of school management, catering firm, dietetic professionals. Policy should focus on reducing fat, saturated fat, sodium content and meeting energy-nutrient requirements of school aged children.

## INTRODUCTION

School foods should be consistent with healthy eating messages.^1^ School Nutrition Programs may have positive effects on children’s health status through key food groups.^2^ Aim of SNP is providing high nutritional quality meals to counteract possible nutrition deprivation at home.^3^ School canteens may have negative impact on health by serving rarely, inadequate fruits-vegetables^4^, foods-beverages rich in energy, fat, carbohydrate, sodium.^5^ SNPs positively affect children’s nutritional intake compared with those who do not eat school meals.^6^ At the beginning of 20^th ^century, USA, Belgium, France, Portugal, Japan, England, Spain constituted school health-nutrition programs.^7^^,^^8^ After first implementations (1956) in Turkey by delivery of milk at schools till 1975^9^, Turkish Government introduced food-based standards legislation for schools (2007) aiming strengthening healthy eating conscious, preventing nutrition related diseases by SNPs.^10^ While school managements tried to establish SNPs, Baş^11^ found that schools in Ankara served adequate energy, protein, high fat, saturated fat. Another city in Turkey was Kayseri in which one of the first governmental schools started serving school lunch. This study was conducted to determine nutritional quality of school lunch and compare lunch consumption of participating and nonparticipating students.

## METHODS

Study was conducted at a Governmental school (Boydak Elementary School) in Kayseri, which served school lunch via catering firm. Sample composed of 116 students aged 9-14 years (11.01±1.74, 48.3% girls). Among 550 attendants, elementary graders who participated in SNP and who could be matched with their nonparticipating fellows were randomly selected. Matching was performed based on grade, gender, age. Participating (SL-G; n=58) and nonparticipating students (SC-G; n=58) in Grades 3-8 were recruited. Students in Grades 1 and 2 could not be included because all should eat school lunch (no matching). Students, who did not attend school during data collection; had lunch from outer canteen; ate home-brought foods for lunch; did not participate in SNP regularly in a whole week, were excluded. Students and parents were informed; verbal consents were taken after institutional approval obtained from Erciyes University Faculty of Health Sciences review committee.

Body weight (digital, sensitive to 100 g scale, King-EB6571, Turkey), height (non-elasting tape) were measured; body mass index (BMI; kg/m^2^) was calculated. Weight, height, BMI for age percentiles were determined.^12^

Energy-nutrient content of 5-day school lunch and lunch consumption of students were analysed. Standardized recipes were used which were served by the catering firm. Weekly menu was fixed-rotated including three dishes with “no-options-for-choice” which was the representative of whole semester weekly menus (Table-I). Socio-demographic data were collected by face-to-face questionnaire. Lunch food consumption was determined by weighing plate waste.^13^ Before service, portion weights of all dishes-beverages were measured with kitchen scales (to nearest gram) (TEM EGE-TB, Turkey). Students in both groups were monitored in case of sharing meals; were not allowed. At the end of lunch, students left trays on tables to measure leftovers. Food intake was calculated by subtracting leftover food weight from allocated portion weight. Bread consumption of SL-G was omitted due to lack of monitorization. Table salt was not allowed to SL-G as a school management decision. Lunch sodium content was obtained by recipes. Lunch consumption of SC-G was recorded concurrently. Portion weights were recorded from food-beverage labels or by weighing unpackaged pastry, doughnut. Wasted foods by SC-G were weighed. Software was used for energy-nutrient data (Ebispro for Windows, Germany; Turkish Version/BeBiS 7).

Adequacy of school lunch was evaluated based on that energy-nutrient content of school lunch should meet 33% of daily requirements of school aged children.^14^ Requirements were based on Dietary Guidelines for Turkey.^15^ To determine adequate consumption, lunch energy-nutrient intakes were compared with 33% of requirements.

Data were analysed by SPSS 15.0. Shapiro-Wilk test was performed to determine normal distribution of data. Student’s t, Mann Whitney U tests were used to compare groups. p<0.05 was set as statistically significant.

## RESULTS

Students (n=116) were assigned to SL-G (n=58) and SC-G (n=58). High percentage of mothers (85.4%) and fathers (98.3%) graduated from college, mostly mothers were housewives (38.8%), fathers lectured at universities (45.7%). Socio-demographic characteristics of two groups were similar.

School lunch menu was examined; mean level (%) of meeting students’ energy-nutrient requirement was calculated (Table-II). Mean energy content was 572±186 kcal; 16.7±4.4%, 46.7±7.5%, 36.6±6.8% was from protein, carbohydrate, fat, respectively. Saturated fat contributed to 12.2±3.5%. School lunch met standard for few nutrients (vitamins E, B_1_, C, fibre, phosphorus, iron). Mean energy, folate, potassium, calcium content was below 33% of requirements (inadequate), vitamins B_2_, B_6_, sodium, magnesium, zinc amounts exceeded lunch requirements (>33%), (Table-II).

Mean level (%) of meeting daily requirements was compared to determine actual energy-nutrients consumption (Table-III). Level of meeting requirements of SL-G ranged from 10.5% (potassium) to 52.5% (sodium) and from 13.2% (folate) to 31.8% (vitamin B_6_). Level of meeting requirements of SC-G ranged from 6.2% (potassium) to 29.7% (sodium) and from 7.6% (folate) to 47.2% (vitamin E). Level of meeting requirements for protein, B vitamins, vitamin C, potassium, iron, zinc consumption of SL-G was significantly higher than SC-G (p<0.001 for each). In SC-G, level of meeting requirements for energy (p<0.001), carbohydrate (p<0.001), fat (p<0.05), fibre (p<0.05), vitamin E (p<0.001) consumption was higher than SL-G (Table-III).

Mostly both groups were normal according to body weight (67.3 and 67.3%, respectively) and BMI for age percentiles (63.9% and 55.2%, respectively), (p>0.05). However, totally 30.1% of students were overweight (10.3%) and obese (19.8%).

## DISCUSSION

The finding that school lunch did not meet lunch energy-nutrients requirements is of great concern for the authors like others.^1^^,^^3^ Energy content of school lunch was lower than other studies^12^^,^^16^^,^^17^ alongside being nearly adequate for relevant sample (28% of requirement). Contributions of fat (35.3% vs. 30%) and saturated fat (12.2% vs. <10%) to energy were high which may be one of the contributors to overweight/obesity in students. Although reducing fat-saturated fat is a priority for school meals^18^, saturated fat content is generally high and 35% fat contribution to energy is still acceptable.^19^ Therefore, serving low-fat dairy products may be enough in reducing fat-saturated fat content of school lunch in this study.

School lunch was not nutrient-dense enough because nutrients were in excessive or inadequate amounts. This may be mainly due to not having food-based or nutrient-based foodservice standards in Turkey which could be adequately applied in all schools, getting foodservice from a catering firm which does not employ a dietitian who is the authority of planning adequate and balanced menu models, and may result from inexperience at school because it was the first one. Although sodium content of bread and table salt was not included, school lunch contained 1001.8 mg sodium due to high addition during cooking. This was even lower than amounts ranging from 1442-1541 mg of lunches in other studies.^17^^,^^20^ Salt amount of school lunches is generally over 80% of recommended intake.^21^ However, it is certain that mean sodium content in this study was still higher than recommendations (≤800 mg)^22^ being a threat for children’s adulthood health.

**Table-I:** School Lunch Menu Served in SNP and Foods-Beverages Consumed by SC-G

*School Lunch Menu*	
*Weekdays*	*Menu Items*
Monday	Lentil Soup Chicken Schnitzel (French Fries) Milk Pudding with Coconut
Tuesday	Cauliflower with Minced Meat Cheese Patty Cold Stewed Quince
Wednesday	Meatball with Potatoes Spaghetti with Tomato Sauce Tomato Salad
Thursday	Kebab with Vegetables Rice Pilaf Yoghurt
Friday	Tomato Soup (Cheese) Meatball (Mashed Potatoes) Ashura
*Foods-Beverages Consumed by SC-G*	
Pastry, Scone, Bagel, Pretzel, Wafer, Cookies, Cakes, Chocolates, Ice-Cream, Milk, Chips, French Fries, Fruit Juice, Soft Drinks	

**Table-II:** Amounts and Level of Meeting Daily Requirements for Energy-Nutrients in School Lunch Menu

*Energy-Nutrients*	*Amount* $\bar{X}$ *± SD*	*Level of Meeting* *Daily Requirements (%)* $\bar{X}$ *± SD*
Energy (kcal)	572±186	28±3.6
Protein (g)	21.7±2.8	48.1±4.4
Carbohydrate (g)	66.6±28.3	42.2±3.5
Fat (g)	23.7±9.6	35.3±4.8
Cholesterol (mg)	50.9±21.7	16.9±3.2
Fiber (g)	6.9±3.9	27.9±7.1
Carotene (μg)	294.6±133.2	51±2.6
Vitamin E (mg)	3.2±2.2	33.6±2.7
Vitamin B_1 _(mg)	0.3±0.1	32.9±1.6
Vitamin B_2 _(mg)	0.3±0.1	43.1±2.8
Vitamin B_6 _(mg)	0.4±0.2	49.9±1.2
Folate (μg)	66.6±18.9	23.5±1.6
Vitamin C (mg)	22±10.6	31±2.3
Sodium (mg)	1001.8±117.8	66.8±4.0
Potassium (mg)	771.8±252.9	17.1±2.0
Calcium (mg)	158.6±87.5	14.4±1.3
Magnesium (mg)	91.5±48.7	46.6±2.5
Phosphorus (mg)	335.9±95.9	38.6±2.7
Iron (mg)	4.1±2.3	39.4±2.4
Zinc (mg)	3.6±0.7	45.7±3.6

**Table-III:** Level of Meeting Daily Requirements for Energy-Nutrient Consumption of SL-G and SC-G

*Energy-Nutrients*	*Level of Meeting * *Daily Requirements (%)*		*t/U*	*p*
	*SL-G (n=58)* $\bar{X}$ *± SD*	*SC-G (n=58)* $\bar{X}$ *± SD*		
Energy	20±5.2	27±11.2	U=746.00	<0.001
Protein	30.7±8.8	16.7±6.1	t=10.00	<0.001
Carbohydrate	28.5±2.9	34.3±3.8	U=187.00	<0.001
Fat	25.9±7.1	30.3±8.7	t= -2.92	0.004
Cholesterol	12.1±2.5	15.5±2.2	U=1339.00	0.058
Fibre	18.1±5.8	21.9±8.7	U=1212.00	0.009
Carotene	28±9.9	26±10.5	U=1536.00	0.420
Vitamin E	24.6±10.5	47.2±25.1	U=712.00	<0.001
Vitamin B_1_	21.1±6.1	13.2±6.4	U=535.00	<0.001
Vitamin B_2_	26.2±8.8	24.9±17.8	U=1341.00	0.059
Vitamin B_6_	31.8±10.5	14.5±6.5	U=211.50	<0.001
Folate	13.2±4.4	7.6±2.9	U=438.00	<0.001
Vitamin C	21±9.7	10±4.9	U=293.50	<0.001
Sodium	52.5±22.0	29.7±10.3	U=334.00	<0.001
Potassium	10.5±2.6	6.2±1.4	U=261.50	<0.001
Calcium	8.3±3.5	9.9±4.7	U=1365.00	0.080
Magnesium	29.2±15.8	19.6±10.4	U=764.00	<0.001
Phosphorus	23.6±11.8	22.2±14.2	U=1333.00	0.054
Iron	21.3±4.8	17.5±5.4	t=3.92	<0.001
Zinc	28.8±11.9	12.9±6.3	U=292.50	<0.001

*t: Student’s t test, U: Mann Whitney U test*

School lunch nearly met standard for vitamin C (31%), but consumed amount by SL-G was inadequate (21%) because the highest wasted dishes were including vegetables; “kebab with vegetables (38.80±3.84% unconsumed)”, “cauliflower with minced meat (48.72±4.30% unconsumed)”. Similarly, few students’ lunch consumption met lunch standards of NSLP; elementary students participating in SNP wasted more than 1/3 of fruit-vegetables, middle school students left nearly 50% of fresh fruit unconsumed.^23^ Vitamin C, folate, potassium intake of SC-G was less than SL-G due to lack of fruits-vegetables in canteen being controversy to legislation.

Studies have shown that SNP participants had favourable protein,^24^ vitamin C, potassium^24^^,^^25^, vitamin A, calcium, iron intakes.^26^ In this study, SC-G consumed significantly more energy, fat, carbohydrate, vitamin E due to eating mainly fried foods, pastry. SL-G consumed significantly higher protein because of meat-legumes which contributed to higher iron and zinc intakes. Despite being statistically insignificant and extremely inadequate; calcium consumption of SC-G was higher due to ayran (yoghurt drink), high wastage of yoghurt by SL-G and rarely serving dairy products in SNP. In Puerto Rico, NSLP participants met requirements for energy from protein, iron, zinc while they did not achieve target intakes for energy, energy from carbohydrates, calcium, magnesium, potassium, fibre. Nevertheless, participant students were concluded to have healthier intake of several nutrients than non-participants.^27^ Despite heal their lunch intake of SL-G, our legislation should be specialized to declare serving amounts of food groups as milk-dairy products, meat-alternatives, fruits-vegetables, starchy foods in SNP. Health Ministry published a guidebook^28^ including menu models being another beneficial step closing us to well-designed SNPs.

Given that obesity rates more than quadrupled among children over past four decades^29^, concerns are expressed on school meals/lunches that they may contribute to obesity.^4^ However, no evidence was found that SNP participation was related to increased overweight/obesity risk.^19^ Significant difference was not determined in body weight and BMI between groups like other researches.^19^^,^^30^^,^^31^ Another recent study does not support a relationship between school-meal participation and BMI.^32^ It cannot be claimed that differences in school lunch energy intake was responsible for overweight/obesity.

Foodservice at this school should be revised by school management, catering firm, dietetic professionals. Efforts are needed to increase availability and accessibility of healthy foods to schools and educate children on accurate food choices by dieticians. Future policy should focus on reducing levels of fat, saturated fat, sodium, increasing fibre, calcium and making school lunches consistent with energy-nutrient requirements of children.

## Notes

***Source of funding:*** None.
